# Pharyngeal Electrical Stimulation for Treatment of Dysphagia in Subacute Stroke: A Randomized Controlled Trial

**DOI:** 10.1161/STROKEAHA.115.012455

**Published:** 2016-05-23

**Authors:** Philip M. Bath, Polly Scutt, Jo Love, Pere Clavé, David Cohen, Rainer Dziewas, Helle K. Iversen, Christian Ledl, Suzanne Ragab, Hassan Soda, Anushka Warusevitane, Virginie Woisard, Shaheen Hamdy

**Affiliations:** From the Stroke Trials Unit, Division of Clinical Neuroscience, University of Nottingham, United Kingdom (P.M.B., P.S.); Phagenesis, Ltd, Manchester Science Park, United Kingdom (J.L.); Centro de Investigación Biomédica en Red de Enfermedades Hepáticas y Digestivas (Ciberehd), Hospital de Mataró, Universitat Autònoma de Mataró, Spain (P.C.); Stroke Service, Northwick Park Hospital, London, United Kingdom (D.C.); Department of Neurology, University Hospital Münster, Germany (R.D.); Department of Neurology, Glostrup Hospital and University of Copenhagen, Denmark (H.K.I.); Schoen Klinik Bad Aibling, Bad Aibling, Germany (C.L.); Stroke Service, Poole Hospital, Poole, United Kingdom (S.R.); Neurology Clinic, Bad Neustadt, Germany (H.S.); Acute Stroke Unit, University Hospitals of North Midlands, Stoke-on-Trent, United Kingdom (A.W.); Unité de la voix et de la deglutition, Service ORL CHU de Toulouse, Octogone Lordat Université Toulouse Mirail, France (V.W.); and Centre for Gastrointestinal Sciences, University of Manchester, United Kingdom (S.H.).

**Keywords:** dysphagia, pharyngeal electrical stimulation, randomized controlled trial, stroke

## Abstract

**Background and Purpose—:**

Dysphagia is common after stroke, associated with increased death and dependency, and treatment options are limited. Pharyngeal electric stimulation (PES) is a novel treatment for poststroke dysphagia that has shown promise in 3 pilot randomized controlled trials.

**Methods—:**

We randomly assigned 162 patients with a recent ischemic or hemorrhagic stroke and dysphagia, defined as a penetration aspiration score (PAS) of ≥3 on video fluoroscopy, to PES or sham treatment given on 3 consecutive days. The primary outcome was swallowing safety, assessed using the PAS, at 2 weeks. Secondary outcomes included dysphagia severity, function, quality of life, and serious adverse events at 6 and 12 weeks.

**Results—:**

In randomized patients, the mean age was 74 years, male 58%, ischemic stroke 89%, and PAS 4.8. The mean treatment current was 14.8 (7.9) mA and duration 9.9 (1.2) minutes per session. On the basis of previous data, 45 patients (58.4%) randomized to PES seemed to receive suboptimal stimulation. The PAS at 2 weeks, adjusted for baseline, did not differ between the randomized groups: PES 3.7 (2.0) versus sham 3.6 (1.9), *P*=0.60. Similarly, the secondary outcomes did not differ, including clinical swallowing and functional outcome. No serious adverse device-related events occurred.

**Conclusions—:**

In patients with subacute stroke and dysphagia, PES was safe but did not improve dysphagia. Undertreatment of patients receiving PES may have contributed to the neutral result.

**Clinical Trial Registration—:**

URL: http://www.controlled-trials.com. Unique identifier: ISRCTN25681641.

## Abstract 2

Supplemental Digital Content is available in the text.

Acute stroke is complicated by oropharyngeal dysphagia in 50% of patients; of these, up to 40% remain dysphagic a year later.^1^ Dysphagia is complicated by aspiration, pneumonia, and malnutrition,^2^ and patients need enteral feeding through a nasogastric tube or percutaneous endoscopically introduced gastrostomy tube, which often requires long-term institutional care.^3^ Although dysphagia may be treated using several physical and behavioral techniques, there are no definitive treatments.^4^

Human swallowing has bilateral representation in the cerebral hemispheres with a dominant cortex (unrelated to handedness).^5^ Dysphagia often follows a stroke that affects the dominant swallowing cortex, which is then exacerbated in recurrent strokes. Swallowing is dependent on afferent feedback via bulbar cranial nerves innervating the pharynx, and increased sensory input from the pharynx can drive long-term beneficial changes in the cortical control of swallowing^6^ with functionally relevant reorganization of the swallowing cortex.^6,7^

During development of pharyngeal electric stimulation (PES), a study in healthy volunteers^8,9^ suggested that PES should be delivered at 5 Hz for 10 minutes with an electric current of threshold plus 75% of the difference between threshold and tolerance levels, a paradigm that produced the largest effect on brain excitability.^8,10^ Using this approach in patients with subacute stroke in a randomized dose-comparison trial, PES reduced radiological aspiration, manifest as a reduction in penetration aspiration score (PAS).^9^ Similarly, PES reduced clinical dysphagia (assessed using the dysphagia severity rating scale [DSRS]) and length of stay in hospital in patients with dysphagia post stroke in a sham-controlled parallel-group phase II trial.^9^ In a further multicentre phase II randomized sham-controlled trial, PES was associated with nonsignificant tendencies to reduced clinical dysphagia and shorter length of stay in hospital.^11^ An individual patient data meta-analysis of these 3 trials found that PES significantly reduced aspiration (PAS) and dysphagia (DSRS) and was safe and well tolerated.^12^ Here, we present the results of a large, randomized, sham-controlled phase III trial of PES in patients with subacute poststroke dysphagia.

## Materials and Methods

### Participants

We did an international, multicenter, randomized, sham-controlled, patient-masked, outcome assessor–masked, parallel-group trial, as detailed in the online-only Data Supplement. In brief, patients with a recent stroke and videofluoroscopy (VFS)-confirmed dysphagia were randomized to 3 days of PES or sham stimulation, and the primary outcome was the penetration aspiration scale, assessed using VFS, at 2 weeks after the third treatment session.

Patients were eligible for the trial if they were admitted to hospital with a clinical stroke syndrome because of ischemic or hemorrhagic stroke, were aged ≥18 years, had clinical dysphagia identified using bedside testing (as assessed by a nurse or speech and language therapist using a local clinical assessment and confirmed by failure on the Toronto Bedside Swallowing Screening Test), were alert or rousable (score of 0 or 1 on question 1a of the National Institutes of Health Stroke Scale [NIHSS]), had a PAS ≥3 (see the online-only Data Supplement for description) of for at least 1 swallow (assessed using VFS),^13^ and could be treated within 42 days of stroke onset. The diagnosis of ischemic or hemorrhagic stroke was confirmed with computed tomography or magnetic resonance imaging performed between hospitalization and enrollment and using standard imaging techniques. Key exclusion criteria included a history of dysphagia, dysphagia from a condition other than stroke, advanced dementia, implanted pacemaker or cardiac defibrillator in situ, unstable cardiopulmonary status or a condition that compromised cardiac or respiratory status, distorted oropharyngeal anatomy, additional diagnosis of a progressive neurological disorder, receiving continuous oxygen treatment, or pregnant or nursing mother.

### Ethics and Approvals

The study was approved by national ethics committees and competent authorities in each participating country, and locally at each site, and was adopted by the UK National Institute for Health Research Stroke Research Network. We obtained written informed consent from each patient, or proxy consent from a relative when the patient did not have capacity (eg, because of dysphasia and confusion), before enrollment and in accordance with national regulations; in Germany, the Bundesamt für Strahlenschutz regulatory authority did not allow proxy consent. The trial was run by a Trial Management Committee (P.M.B., S.H., C.M., and J.L.). An independent data-monitoring committee reviewed unmasked data every 6 months. The trial was registered as ISRCTN25681641.

### Randomization

VFS (see below) was performed as a study procedure after consent to confirm the presence of dysphagia (PAS ≥3).^14^ Investigators entered baseline and follow-up data into a commercial database (Rave, Medidata Solutions, Inc) linked to a randomization list (Quantics Consulting, Ltd). The data were checked to confirm the patient’s eligibility, and the system then assigned a participant to treatment with active PES or sham PES with allocation 1:1. Allocation was by randomly permuted blocks (of size 6) with stratification by center and feeding status (presence/absence of artificial feeding) to enhance balance between treatment groups.

### VFS

VFS was performed using local protocols at each participating site by a speech and language therapist or a radiologist. At each time point (baseline and weeks 2 and 12), each participant was given up to 6×5 mL bolus drinks of contrast agent (Omnipaque 300 in UK, Visipaque 270 in France, or Accupaque 300) of liquid consistency (≈40% wt/vol). A 50 mL drink of contrast agent was then administered and swallows recorded.

At baseline, bolus drinks were taken until 3 were positive (ie, at least 1 swallow within a bolus of PAS ≥3); once achieved, further bolus drinks were not given to reduce the risk of aspiration and pneumonia. Hence, between 3 and 7 boli (each inducing ≥1 swallows) were administered. Once completed, quality-assured digital VFS image files for each swallow for each bolus were sent immediately to 1 of 2 independent adjudicators who were blinded to clinical information and who confirmed whether the patient fulfilled the inclusion criteria on the basis of aspiration of radiological contrast. Use of digital VFS reduced the risk of image degradation on file transfer. Once confirmation was received, treatment could be started. VFS images at weeks 2 and 12 were similarly uploaded and assessed by 1 of 2 adjudicators who were blinded to patient details and randomization. Silent aspiration was defined as aspiration without an attempted cough as seen on the video file, accompanying sound, or event monitor.

### Procedures

Sterile single-patient use treatment catheters (Phagenyx, Phagenesis, Ltd, Manchester, UK), which contain an inner lumen for feeding, were inserted via the nose by trained staff. The catheter was inserted to an aboral depth related to the patient’s height so that the pair of ring treatment electrodes located on the outer surface of the catheter were adjacent to the pharynx.

Treatment was started once dysphagia was confirmed by VFS and given daily for 3 days.^9^ At each session, the catheter was connected to the controlling base station, and electric current at 5 Hz was increased incrementally from 1 mA to detect threshold (patient first aware of stimulation) and then tolerated (patient does not want current increased further) intensity levels in all patients. Those randomized to active PES were then administered this for 10 minutes at a treatment current (mA) of threshold plus 75% of the difference between threshold and tolerance levels; this paradigm was used successfully in earlier studies of PES and considered to be an effective level of stimulation without being too near the tolerance level.^12^ Patients randomized to sham therapy had no stimulation after establishment of threshold and tolerated levels. Patients, but not the treating researcher, were masked to treatment assignment. Treatment could be stopped if the patient withdrew consent, for safety reasons, or if unacceptable adverse events developed.

Active or sham PES treatment was given in addition to standard stroke care, including thrombolysis if administered at admission to hospital, and rehabilitation. Systematic use of antihypertensive agents (all patients), oral antithrombotic and lipid-lowering agents, and carotid endarterectomy (patients with ischemic stroke) were recommended for secondary prevention as per each site’s local practice. The final diagnosis was confirmed at discharge based on clinical presentation and neuroimaging.

### Outcomes

The primary outcome measure was radiological aspiration at 2 weeks assessed as the PAS using VFS.^14^ The timing of VFS at 2 weeks reflected that used in 3 pilot trials.^12^ As a secondary outcome, PAS was also measured at 12 weeks.

Other prespecified secondary outcomes at 2, 6, and 12 weeks included clinical dysphagia (DSRS^9^; see the online-only Data Supplement), dependency (modified Rankin Scale [mRS]^15,16^), activities of daily living/disability (Barthel Index^17^), impairment (NIHSS^18^), health-related quality of life (European Quality of Life-5 Dimensions [EQ-5D],^19^ from which health utility status was calculated [EQ-5D-HUS]), and nutritional measures (weight, mid-arm circumference, and blood albumin). At discharge from initial admittance to hospital, investigators recorded duration of stay and discharge destination (to institution or home).

The safety outcomes were all-cause case fatality and cause-specific case fatality; serious adverse events and serious adverse device-related events; and cases of chest infection or pneumonia (diagnosed locally because the diagnosis of chest infection and pneumonia is poorly defined^20^).

A member of the central research team (S.H.), who was masked to treatment assignment, validated and categorized investigator-reported serious adverse events, including cause-specific deaths. Patients who did not receive their assigned treatment or who did not adhere to the protocol were followed up in full. The recruiting site, using a separate nontreating researcher who was masked to treatment allocation, did post-treatment follow-ups at 2, 6, and 12 weeks.

### Statistical Analyses

The statistical analysis plan was published on the Phagenesis, Ltd, website before data lock and unblinding: http://www.phagenesis.com/wp-content/uploads/2012/09/Statistical-Analysis-Plan-STEPS.pdf (March 21, 2012). The trial was designed to recruit 140 patients so as to detect an absolute reduction in the change in PAS (mean of all swallows from all available boli) from baseline to 2 weeks of 1.1 point (SD 1.8) between the treatment groups, with power 90%, 2-sided significance 5%, and allowance for incomplete data/losses to follow-up in 15% of patients. After analysis of individual patient data from 3 pilot studies,^12^ the primary analysis was changed to comparison between the treatment groups of the mean of the worst swallow in each of the 3 to 7 available boli (with adjustment for the same at baseline, and no imputation of missing data) because this seemed to be more robust statistically and was felt to be clinically more relevant, a decision that was made before unblinding of data.

Four analysis populations were created: randomized, all those who were assigned to PES or sham treatment; safety, all randomized patients who had treatment attempted, that is, insertion of the treatment catheter with or without PES/sham; efficacy, all randomized patients who received at least 1 episode of PES/sham treatment and who had the primary outcome (PAS) measured at both baseline and 2 weeks; and per protocol, randomized patients who received all 3 treatments and who had PAS data measured at baseline and 2 weeks.

Swallowing was analyzed as a comparison between the treatment groups using multiple linear regression with adjustment of the on-treatment PAS for baseline PAS, stratification variables (site and feeding status), and prognostic baseline variables (age, sex, and NIHSS). Secondary analyses used multiple linear regression (continuous data, eg, EQ-5D), ordinal logistic regression (ordered categorical data, eg, mRS), binary logistic regression (dichotomous data, eg, PAS ≤3, serious adverse events, and chest infection), and Kaplan–Meier and Cox regression models (time to event, eg, death). 95% confidence intervals (CI) are presented, and *P*<0.05 was considered statistically significant. Analyses were performed using SAS version 9.3. Summary meta-analyses based on group data from Swallowing Treatment Using Pharyngeal Electrical Stimulation (STEPS) and earlier trials^9,11^ were produced using the Cochrane Collaboration’s Review Manager software (version 5.3).

### Additional Information

Further information on Materials and Methods is given in the online-only Data Supplement.

## Results

**Figure 1.**
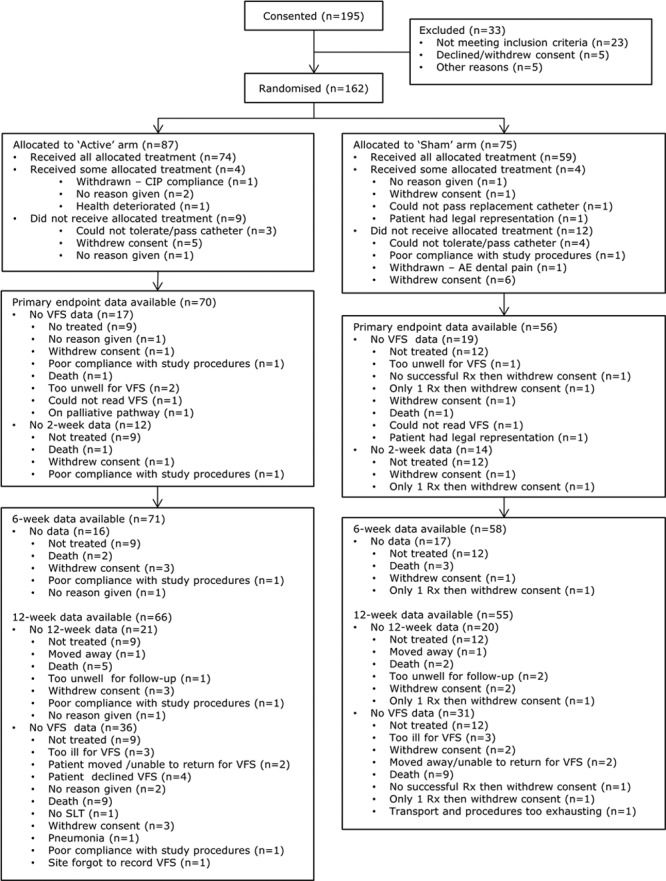
Flow of patients through the trial: consented, 195; screened with VFS, 181; randomized, 162; treatment attempted, 152; treated, 141; treated with VFS at 2 weeks, 126; all 3 treatments received with VFS at 2 weeks, 123; treated with VFS at 12 weeks, 95. AE indicates adverse event; CIP, clinical investigational plan; Rx, randomization; SLT, speech and language therapy; and VFS, videofluoroscopy.

Between April 2012 and September 2014, we consented 195 patients; screened 181 patients with VFS; assigned treatment in 162 patients (randomized population); attempted treatment in 152 patients (safety population); treated (with at least 1 session of PES or sham) 141 patients; and obtained VFS in 126 patients at 2 weeks (primary outcome population) and 95 patients at 12 weeks (Figure 1). The reduction in numbers between consent and randomization reflected patients who: screened negative for aspiration on VFS, could not have the catheter inserted, and did not have a VFS 2 weeks after treatment. The 162 randomized patients were recruited from 20 sites in 5 countries (Denmark, France, Germany, Spain, and United Kingdom, listed in the online-only Data Supplement); of these, 87 patients were assigned active PES and 75 patients were assigned to the sham group (Figure 1). Hundred and one patients (62.3%) were recruited from the United Kingdom. The randomized groups were well balanced at baseline (Table 1): mean age 74 (SD 11) years, 94 (58%) were male, and 143 (89%) patients had an ischemic stroke. The mean time from stroke to randomization was 13 (10) days. The Data Monitoring Committee reviewed the trial on 3 occasions and recommended that the trial should continue each time.

**Table 1.**
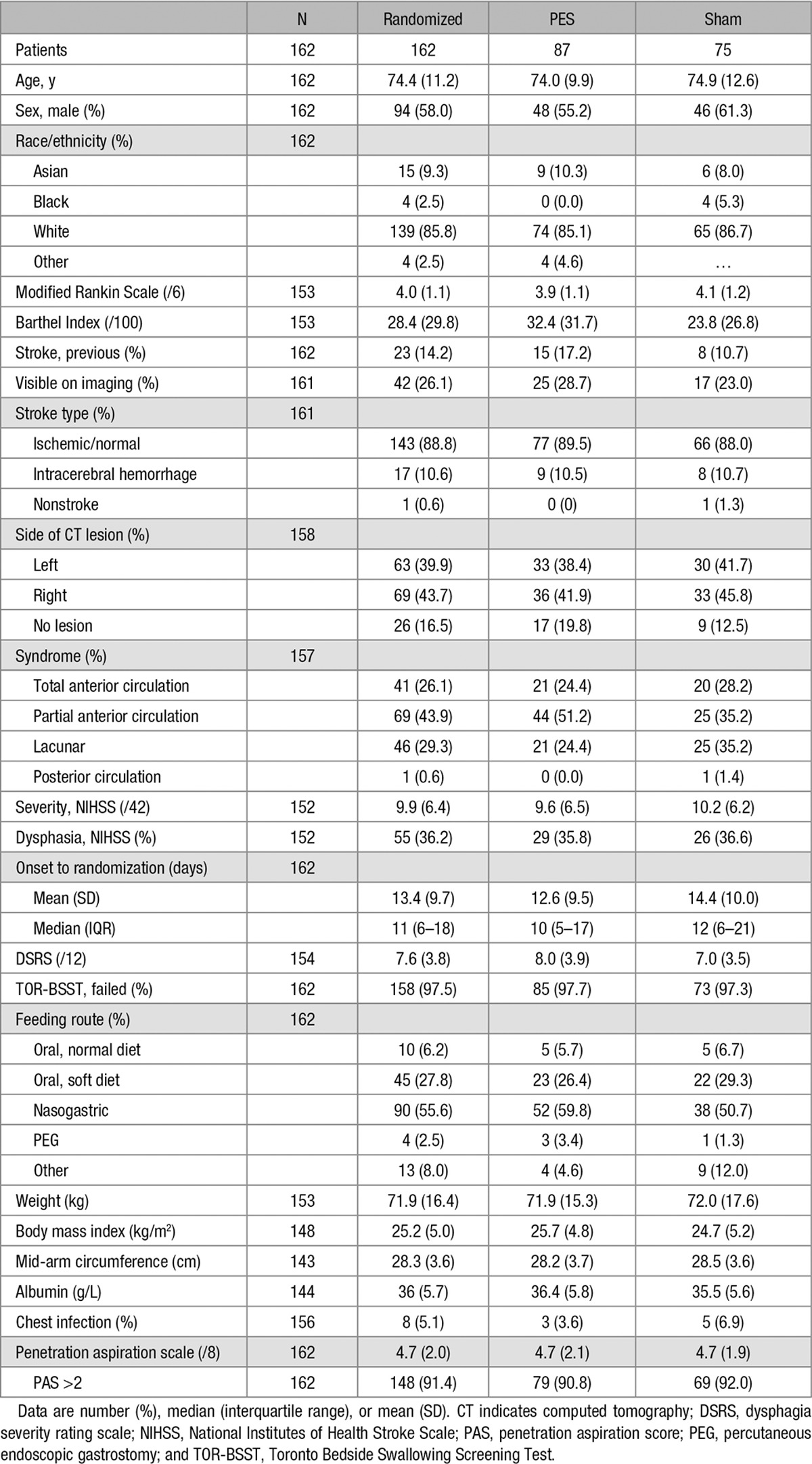
Baseline Characteristics in the Randomized Population by Treatment Assignment

Adherence with assignment to active or sham PES was good in 141 participants who received at least 1 treatment session. There were no material differences at baseline in 15 treated participants who did not have VFS at 2 weeks versus 126 treated participants who did have VFS. No patients randomized to sham received active treatment, and all patients with a catheter inserted and randomized to PES received at least 1 active treatment session. The mean treatment stimulation level was 14.5 mA in those randomized to PES, with mean treatment duration 9.8 minutes and mean number of treatments 3.0 (Table I in the online-only Data Supplement). However, evidence of suboptimal treatment current levels seemed to be present: 58% of PES-treated patients had a treatment level <10.2 mA (a figure chosen from earlier research^12^), identical treatment and threshold levels, or a treatment level less than threshold.

**Figure 2.**
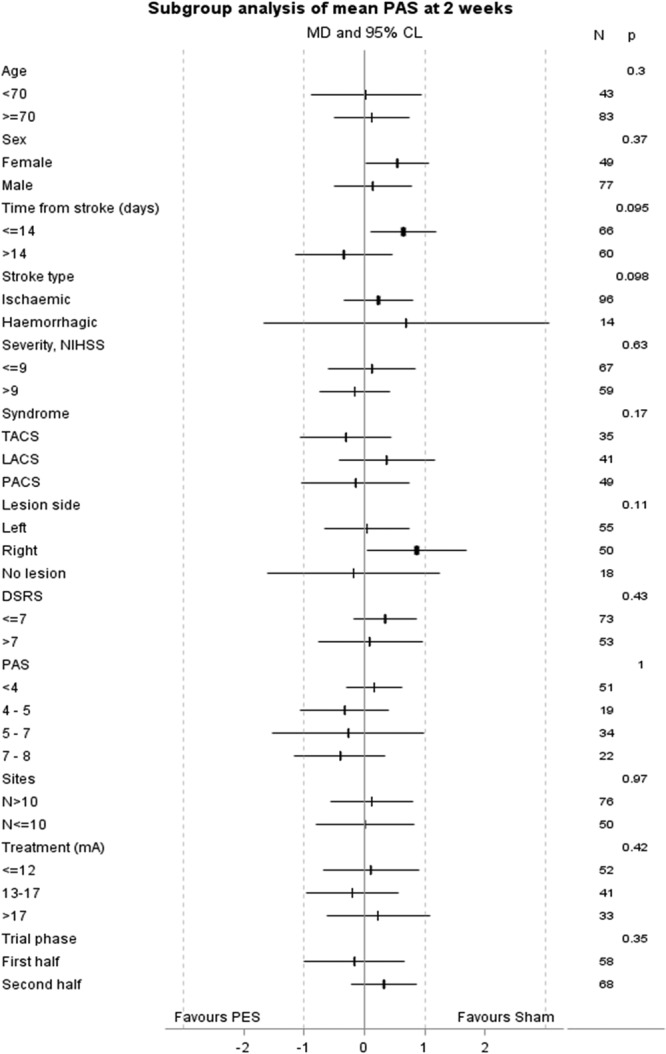
Effect of treatment on penetration aspiration score in prespecified subgroups determined at baseline, with analysis using adjusted multiple linear regression. CL indicates confidence limit; DSRS, dysphagia severity rating scale; LACS, lacunar circulation syndrome; MD, mean difference; NIHSS, National Institutes of Health Stroke Scale; PACS, partial anterior circulation syndrome; PAS, penetration aspiration score; PES, pharyngeal electric stimulation; and TACS, total anterior circulation syndrome.

In the primary outcome population, the mean PAS at baseline was 4.8 (SD 2.0) and reduced in both active PES and sham PES groups at 2 weeks (Table 2). When adjusted for site, age, NIHSS, baseline feeding status, and PAS, there was no difference in PAS at 2 weeks, mean difference 0.14 (95% CI, −0.37 to 0.64; *P*=0.60; Table 2 and Figure 2); the mean change in PAS from baseline to 2 weeks did not differ between the 2 treatment groups: active PES −1.2 (1.8) versus sham PES −1.2 (1.8) and difference 0.14 (−0.37 to 0.64). Meta-analysis of individual patient data from earlier studies suggested that different approaches to statistical analysis varied in their statistical efficiency;^12^ in sensitivity analyses, PAS did not differ between the groups when assessed using different statistical approaches (Table II in the online-only Data Supplement). When assessed in prespecified subgroups, no significant interactions were present (Figure 2).

**Table 2.**
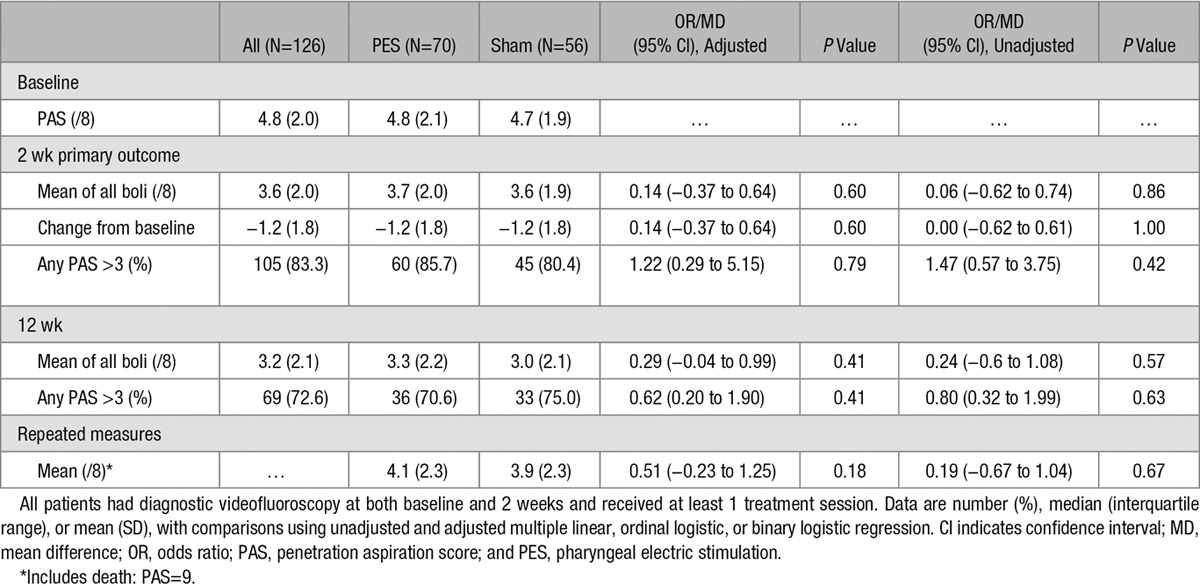
PAS at 2 Weeks in the Efficacy Population by Treatment Assignment

PES had no significant effects on secondary measures of swallowing and feeding, including radiological aspiration (PAS) at 12 weeks, and clinical dysphagia (DSRS) and feeding route at weeks 2 and 12 (Table 3; Table II in the online-only Data Supplement). Apparent tendencies in favor of PES were present at week 2 (but not at week 12) for functional measures of outcome (mRS and Barthel Index). Other measures did not differ between the treatment groups (Table 3; Table II in the online-only Data Supplement). When assessed in prespecified subgroups, significant interactions were present between clinical dysphagia (DSRS) and treatment assignment for age and PAS (Figure I in the online-only Data Supplement). The number of patients with chest infection or pneumonia occurring after randomization (and so possibly related to VFS rather than subsequent PES/sham treatment) did not differ between the treatment groups: PES 21, sham 11 (*P*=0.19). The overall rate of serious adverse events occurring by end of follow-up did not differ between the 2 groups, and no serious adverse device-related events occurred in either group (Table III in the online-only Data Supplement). The cumulative risk of all-cause death during follow-up did not differ between the group given PES and the sham treatment (Figure II in the online-only Data Supplement). The treatment equipment was rated as easy to use by investigators who operated the PES treating device; however, passing the catheter was rated as difficult in one third of investigators (Table IV in the online-only Data Supplement).

**Table 3.**
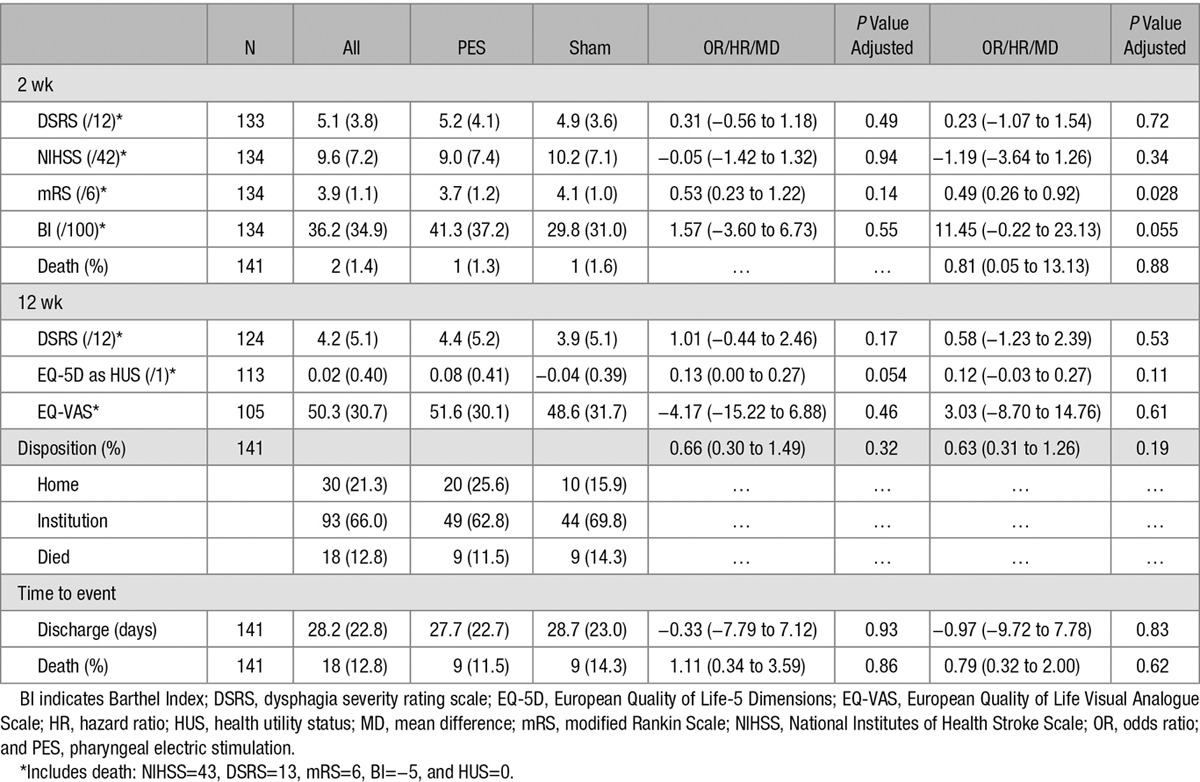
Clinical and Safety Outcomes by Treatment Assignment in Patients Who Received At Least 1 Active or Sham Treatment and Who Had Outcome Measured

In a summary meta-analysis of results from STEPS and earlier trials,^9,11^ there was no difference in PAS between patients randomized to PES versus sham (Figure III in the online-only Data Supplement). In contrast, PES was associated with a larger reduction (ie, improvement) in DSRS than patients randomized to sham, mean difference −0.94 (95% CI, −1.85 to −0.03; *P*=0.04; Figure IV in the online-only Data Supplement).

## Discussion

In patients with dysphagia post stroke, PES had no significant effect on radiological aspiration or clinical dysphagia, assessed as PAS and dysphagia severity rating scale, respectively. Similarly, PES had no effect on dependency (mRS), disability (Barthel Index), or impairment (NIHSS). No safety issues were identified.

The explanation for these largely neutral results remains unclear but many possibilities need to be examined. First, PES may simply not be effective for treating dysphagia after stroke; however, this seems unlikely in the context of a positive individual patient data meta-analysis of earlier poststroke PES studies,^9,11,12^ the positive summary meta-analysis for DSRS presented here, and positive trials in multiple sclerosis and stroke patients with a tracheostomy.^21,22^ Second, the severity of dysphagia at baseline will itself determine the likely success of treatment. Across the field of acute stroke, it is challenging to demonstrate efficacy in a group of patients with mild impairment because many patients will regain normal function spontaneously; in this context, mild dysphagia is likely to resolve spontaneously. Importantly, the regulatory authority in 1 country (Germany) limited recruitment to patients who could provide consent for themselves, and this resulted in inclusion of patients with only milder stroke and aspiration, a decision that would challenge demonstrating efficacy for many interventions. Although the mean baseline PAS in STEPS (PAS=4.8) was similar to previous stroke trials of PES (4.3^12^; Table V in the online-only Data Supplement), it was lower than in a positive trial in multiple sclerosis (PAS=6.5^21^). Of relevance, patients randomized to sham in the earlier studies tended to have minimal or no overall improvement in PAS or DSRS, whereas sham patients in STEPS showed improvement (Table V in the online-only Data Supplement). Confounding this point is the potential relevance of VFS to the diagnosis of dysphagia and its severity; in particular, PAS scores were noted to be highly variable during administration of contrast boli. Additionally, VFS was not readily available at many sites thereby limiting recruitment. We chose PAS (using thin boli) as a primary outcome measure based on previous pilot studies which showed a significant improvement in this measure in the active PES arm^8,9^ but recognize that PAS alone does not capture information about swallowing efficiency and bolus control as might come from using thick liquid boli and measures of pharyngeal residue/timings.

Third, and related to the issue of severity and spontaneous resolution, patients who are enrolled early after stroke will comprise a mixed group of those with severe dysphagia and those with milder dysphagia that will improve without treatment. However, later recruitment will enhance the proportion of patients with severe (or fixed) dysphagia. In reality, STEPS and earlier trials each recruited patients at ≈2 weeks poststroke.^12^ Fourth, participants received variable amounts of active speech and language therapy, and this may have confounded the effect of additive PES.

Fifth, patients randomized to PES may have received subtherapeutic stimulation levels because mean levels were lower in STEPS (mean treatment 14.8 mA) than in previous positive trials in stroke (16.8 mA^12^). Using a treatment level of <10.2 mA (mean − 1 SD in previous trials^12^) or treatment threshold level ≤0 mA, 58% of participants randomized to PES may have been undertreated. Importantly, the magnitude of stimulation has been shown previously to be associated with improvement in aspiration.^8^ Investigator concerns about the potential to harm patients seem to have explained this situation, although the study showed no evidence of harm, and PES may be delivered safely up to 50 mA (the maximum that can be delivered by the base station), as shown in another study in patients with stroke.^22^ And last, assessment of threshold and tolerance levels in patients randomized to sham PES may have amounted to an element of stimulation. For example, a participant randomized to sham but who had high threshold and tolerance currents will have received a potentially therapeutic form of stimulation for 10 to 20 minutes (as compared with the 30+ minutes that patients randomized to active treatment receive). These potential explanations for the STEPS results have implications for the design of future trials of PES (and, indeed, other device trials) and training of investigators.

STEPS has several strengths, including the large sample size relative to previous studies of PES; generalizability because of wide inclusion criteria with both ischemic and hemorrhagic stroke, cortical, lacunar, and posterior syndromes, and a wide time window; recruitment from multiple countries in Europe; central concealment of treatment assignment; prospective collection of multiple aspiration, dysphagia, functional, and safety outcomes; and quality care in stroke units.

However, several limitations are also present. First, 195 patients were consented, 162 patients randomized but only 126 received at least 1 treatment session and had both a baseline and on-treatment PAS. Several factors explain this dropout, including withdrawal of consent and failure of insertion of the treatment catheter (Figure 1). A protocol amendment required that the treatment catheter had to be inserted before, and not after, randomization to reduce losses of patients who were randomized but could not be treated. Second, PES was delivered in 141 patients but 15 could not have VFS performed at both baseline and week 2 thereby excluding them from the primary analysis. Third, PES was given in a single-blind design with the patient but not treating person masked to stimulation. Some patients receiving active PES may have been aware of stimulation, whereas patients randomized to sham PES may have been aware of stimulation during threshold testing and possibly noticed that this was absent during the treatment sessions. Nevertheless, clinical outcomes measured at 2, 6, and 12 weeks were assessed by trained staff who were masked to treatment assignment and who were not involved in hospital care of enrolled patients. Furthermore, VFS images were adjudicated by radiologists or speech therapists who were similarly masked to randomized group.

In conclusion, we found that PES did not reduce radiological aspiration or clinical dysphagia. This result differs from a positive meta-analysis of previous small trials of PES in poststroke dysphagia^12^ and may result from several factors, including enrollment of patients with mild dysphagia, potential undertreatment with PES, and possible active stimulation of control patients. In view of this discrepancy, and the potential risk of overestimating treatment effect from smaller studies, further studies are planned in stroke patients with severe dysphagia or those requiring intensive care including ventilation.

## Acknowledgments

We thank the investigators and research staff at the participating sites for their support and acknowledge the support of the UK National Institute for Health Research, through the Stroke Research Network. P.M. Bath is Stroke Association Professor of Stroke Medicine.

## Sources of Funding

The trial was sponsored and funded by Phagenesis, Ltd (Manchester, UK).

## Disclosures

P.M. Bath received honoraria for work as the Chief Investigator and for consultancy. S. Hamdy is the inventor of PES and has stock in Phagenesis. J. Love was an employee of Phagenesis. Institutions using P. M. Bath, D. Cohen, H.K. Iversen, R. Dziewas, V. Woisard, and P. Clavé received per-patient fees for recruitment. P.M. Bath, P. Scutt, D. Cohen, H.K. Iversen, R. Dziewas, and V. Woisard received travel expenses for attending meetings. The other authors report no conflicts.

## Supplementary Material
