# Supplementary material for: Pharyngeal Electrical Stimulation for Treatment of Dysphagia in Subacute Stroke: A Randomized Controlled Trial
Source: Stroke. 2016 May 23;47(6):1562–70. doi: 10.1161/STROKEAHA.115.012455 (PMC4878285; doi:10.1161/STROKEAHA.115.012455)
Supplement: Supplementary file 1 [file str-47-1562-s001.docx]

**SUPPLEMENTAL MATERIAL**

**Title**

Pharyngeal electrical stimulation for treatment of dysphagia in subacute stroke: a randomised controlled trial (ISRCTN25681641)

**Writing Committee**

Philip M Bath, Polly Scutt, Jo Love, Pere Clavé, David Cohen, Rainer Dziewas, Helle K Iversen, Christian Ledl, Suzanne Ragab, Hassan Soda, Anushka Warusevitane, Virginie Woisard, Shaheen Hamdy

**Trial Steering Committee**

Philip M Bath (Nottingham, UK), Shaheen Hamdy (Manchester, UK), Conor Mulrooney (Manchester, UK), Jo Love (Manchester, UK).

**Phagenesis Ltd (Manchester, UK)**

Jo Love, Danny Green, Conor Mulrooney, David Drown, Jane Korby, Sian Scriven.

**Data Monitoring Committee**

Martin Dennis (Chair/Stroke Physician; Edinburgh, UK), Ian Ford (Biostatistician; Glasgow, UK), Susan McGowan (Speech and Language Therapist; London, UK).

**Videofluoroscopy Adjudicators**

Elizabeth Boaden (Moore, UK), Roger Newman (Preston, UK), Dana Hadley (Sheffield, UK)

**SAE Adjudicator**

Shaheen Hamdy (Manchester, UK)

**Statistical analysis**

Polly Scutt (Nottingham, UK).

**Participating Countries and Sites**

By country (number of randomising sites, participants) and sites (number of randomised participants): Principal Investigator, other Investigators †National Coordinator

*Denmark (3, 17):*

- Bispebjerg (2): P Meden, E Engemann, A Hansen, S Hamburg, L Ingemann, J Jansen, H Pedersen, S Rosenbaum, S Ulnits
- Glostrup (11): H Iversen †, S Andersen, S Eskildsen, V Fazilati, P Hornslet, S Jensen, B Langhorn, L Petersen, S Simonsen, D Skovgaard, H Sørensen, J Veng-Olsen, P Vögele, A West, T Wienecke
- Herlev (4): C Kruuse, D Hammer, C Hansen, L Molgård, L Nielsen, D Nørvang, V Petrovic, J Tybring

*France (2, 7):*

- Nantes (1): M Le Fort, O Bassard, A Chenet, E Cohen, P Hamon, B Hauraix, P Rapin
- Toulouse (6): V Woisard †, S Bodin, S Crestani, N Fenon, P Fichaux-Bourin, S Grand

*Germany (4, 28):*

- Bad Aibling (9): F Müller, C Ledl, J Doleschal, K Hinterberger, D Jungbluth, Y Knerr, M Lehmann, S Mudungaze, R Oldenburg, K Rieß, B Schäpers, M Schlutt, M Schumacher, K Trautwein
- Bad Neustadt (10): H Soda, B Behringer, S Fodor, A Friede, F Kasprsak, M Kirchner, A Märkert, R Weinhardt
- Bielefeld (1): W Schäbitz, S Basli, K Berends, B Berger, A Bienek, U Grage, C Hagemeister, N Jansen, A Sinning
- Münster (8): R Dziewas †, B Buerke, D Schulte to Bühne, C Hamacher, S Ölenberg, S Suntrup

*Spain (1, 9):*

- Mataró, Barcelona (9): P Clavé †, V Arreola, V Casado, I López, A Martín, L Mundet, D Muriana, E Palomeras, L. Rofes, N Vilardell

*UK (10, 101):*

- Essex (1): G Zachariah, V Biggs, K Blackmore, D Briggs, F Cairns, L Cooper, J Gunn, A Lyle, F McNeela, E Rafferty, S Smolen, K Swan, J Topliffe, V Umachandran, H Walsh, S Walsh, M Warwick
- Exeter (1): M James, A Bowring, N Chivers, R Evans, K Gupwell, G Guscott, S Hayes, S Keenan, H Kingwell, D Parsons, J Short, D Thomas
- Leicester (7): A Mistri, S Anand, P Christian, B Cole, M Dickens, D Everson, C Haunton, S Khan, L Manning, R Marsh, H McQueen, D Shaw
- London, Imperial (3): O Halse, S Amlani, L Bolton, D Brown, S Burkitt, E Catangui, K Collins, T Davies, J Godber, K Harrall, B Hazel, A Lacey, E Lawlor, T Sachs, J Slark, E Talbot, V Tilley, E Wackrow, S Wegecsanyi, L White, P Wilding, J Zimmerli
- Northwick Park (37): D Cohen, S Ackford, R Bathula, L Burgess, R Cave, C Cawley, S Condie, C Cowley, J Devine, M Macfarlane, M Mpelembue, E Tweedie, D Wood
- Nottingham (14): P Bath,† A Buck, J Clarke, P Cox, L Daunt, N Gilzeane, D Havard, JC Jobling, K Krishnan, S Rajakaruna, K Richardson, J Roffe, J Saunders, C Scottow, S Sheikh, F Shelton, L Wilkins
- Poole (18): S Ragab, D Broadbent, E Cowan, C Dickson, J Edington, L Gleave, A Grogan, E Jinks, J Legett, R Middle, U Naghotra, M Price, M Stevenson, L Sykes
- Stoke-on-Trent (15): A Warusevitane, A Barry, J Bee, C Bradbury, J Chenbala, L Dale, P Ferdinand, K Finney, J Grocott, J Hiden, J Lucas, S Lyjko, H Maguire, I Natarajan, M Richardson, C Roffe, R Sanyal, J Weerathunga
- Truro (2): F Harrington, K Adie, G Courtauld, D Gomes, A James, L Lucas, A Mate, P O’Brien, C Schofield, J Skewes, J Wright
- Wolverhampton (3): K Fotherby, N Ahmad, D Butler, C Cresswell, M Haley, K Jennings-Preece, F Lewis, D Morgan

**Authors’ contribution**

All authors contributed to the interpretation of the results and writing of this report. As Chief Investigator, PMB prepared the protocol, supervised and reviewed the progress of the trial, recruited patients, and wrote the first draft of this report. JL prepared the protocol, and supervised and reviewed the progress of the trial. Members of the writing committee supervised and reviewed the progress of the trial, and commented on the draft of this report. PS analysed trial data and commented on a draft of this report. All members of the writing committee have seen and approved the final version of this report.

**Declaration of Interests**

The trial was sponsored and funded by Phagenesis Ltd. PB received honoraria for work as the CI and for consultancy. SH is the inventor of PES, and has stock in Phagenesis. JL was an employee of Phagenesis. Institutions employing PB, DC, HKI, RD, VW, and PC received per patient fees for recruitment. PB, PS, DC, HKI, RD, and VW received travel expenses for attending meetings.

**Acknowledgments**

This trial was funded by Phagenesis Ltd. We thank the investigators and research staff at the participating sites for their support, and acknowledge the support of the UK National Institute for Health Research, through the Stroke Research Network; recruitment in the UK would not have been possible without network support. PMB is Stroke Association Professor of Stroke Medicine.

**SUPPLEMENTAL METHODS**

**Training of Investigators**

All STEPS investigators were trained in the protocol, Good Clinical Practice, and use of the National Institutes of Health Stroke Scale, modified Rankin Scale (mRS) and Barthel Index. Investigators who delivered PES were trained in the technique.

**Schedule for Monitoring of Sites and Data Integrity**

Site monitoring was performed by trained Clinical research Associates who worked for Phagenesis Ltd or a delegated organisation. Their aim was to ensure quality control for the delivery of the protocol, collection of data and adherence with national regulations and ethics. Each recruiting site had a start-up visit for training, a monitoring visit after recruitment of the first patient, and a close-down visit; further visits were performed as deemed necessary by the Company. Monitoring visits confirmed the presence of the participant and their consent, eligibility criteria, 100% of data, and reporting of serious adverse events.

Central statistical monitoring of the data was performed according to Buyse *et al* [**^1^**](#_ENREF_1) prior to locking of the data. Checks included logic and range checks, and digit preference. The monitoring procedures were compliant with the requirements of the sponsor, the national ethics committees and regulatory authorities in the participating countries, and fulfilled Good Clinical Practice requirements.

**Sample Size Considerations**

The primary endpoint for the study was change in mean penetration-aspiration scores (PAS) on the videofluoroscopy protocol, 2 weeks after treatment. The sample size was based on data gained from a phase II sham-controlled study.[^2^](#_ENREF_2) In this study, the mean values of the change in mean PAS in the two randomised groups of sizes 16 and 12 were -1.4 and -0.1, giving an observed treatment effect of 1.3.The standard deviations in each group were 1.9 and 1.5 respectively. The distributions of values in each group were approximately normal, with standard deviation of 1.8.

Sixty patients in each group would provide 90% power to detect a difference of approximately 1.1 in the change in mean PAS, based on comparison of change in mean PAS between the two groups using a 2 sided t test, with alpha = 5%. The investigation was expected to provide higher power than this because the primary endpoint was adjusted for severity at baseline. To account for the loss of information caused by the potential for less than six swallows being available for every patient, and to account for patients dropping out before the two-week assessment, the sample size was increased by approximately 30%.

Therefore, 160 patients should be randomised in a 1:1 ratio between the treatment groups, this allowing for a one-in-six attrition rate at 2 weeks.

**Independent Data Monitoring Committee (IDMC)**

The IDMC was responsible for safeguarding the interests of trial patients, assessing the safety and efficacy of the intervention during the trial, assessing data integrity, and for monitoring the overall conduct of the trial. The IDMC *modus operandi* was defined in a charter. The IDMC reviewed the recruitment of patients, and assessed safety and efficacy measures by treatment group. The trial was reviewed on three occasions during the trial’s recruitment period. The DMC was charged with informing the Trial Steering Committee if, at any time, the data showed evidence beyond reasonable doubt of a difference between the randomised groups in the primary outcome or for death. They also considered these data in the light of external information such as results from completed trials. One formal interim analysis was performed after 60 participants had been enrolled and completed the 2-week assessments. However, the DMC could perform statistical comparisons as they deemed necessary, with stopping criteria based on the Haybittle-Peto stopping rule (i.e. a difference of 3 standard errors is considered as clear evidence of a treatment effect). The study was not terminated early.

**Inclusion and Exclusion Criteria**

#### Screening Inclusion Criteria

- Subject is over 18 years of age
- Subject is suspected of having dysphagia
- Subject is able to comply with videofluoroscopy protocol
- Subject diagnosed with stroke, whether anterior or posterior circulation
- Subject has no previous history of dysphagia
- Subjects who are able to give voluntary, written informed consent to participate in the clinical investigation and from whom consent has been obtained/ or a consultee has consented on the subjects behalf in line with nationally agreed guidelines concerning adults unable to consent for themselves.
- Subject is not currently participating in any other interventional clinical study
- Subject is able to comply with CIP requirements
- Subject scores 0 or 1 on questions 1a of NIHSS

#### Randomisation Inclusion Criteria (post consent)

- Subject has confirmed dysphagia (PAS of 3 or more on VFS screening protocol)

#### Exclusion Criteria

- Subject stroke event was more than 42 days ago
- Subject is pregnant or a nursing mother
- Subject, in the opinion of the investigator, has advanced dementia
- Subject fitted with a pacemaker or implantable cardiac defibrillator
- Subject has unstable cardiopulmonary status (e.g. severe emphysema, heart failure)
- Subject has distorted oropharyngeal anatomy (e.g., pharyngeal pouch)
- Subject is dysphagic from conditions other than stroke
- Subject has been diagnosed with a progressive neurological disorder (e.g. Parkinson’s disease, Multiple Sclerosis)
- Subject has a chronic medical condition that compromises cardiac or respiratory status (e.g. severe emphysema or heart failure that may render the insertion of the throat unsafe)
- Subject is receiving continuous oxygen treatment or the equipment for this is in place.

Patients in intensive therapy unit, whether intubated or not, were not included since many would require oxygen treatment.

**Procedure for Videofluoroscopy (VFS, modified barium swallow)**

VFS was performed by a trained and qualified speech & language therapist or radiologist; they had to comply with the STEPS VFS protocol and ‘stop’ criteria. A common VFS protocol was used across all participating sites. Strict adherence is required to this VFS protocol and the criteria to ‘stop’ the procedure as defined below.

*Preparation*

- The subject must remain nil by mouth for 60 minutes prior to the research VFS.
- The person delivering treatment/sham must not be present during the VFS.
- Capture a ‘test frame’ using the x-ray equipment to ensure all data will be captured.
- Ensure VFS study data is anonymised.
- Ensure use of lead numbers or annotation throughout the VFS to identify each trial clearly to the independent VFS analysers.
- Ensure use of the suprahyoid marker throughout the VFS procedure.
- Ensure the full swallow is recorded in a lateral view using continuous/25 or 30 frames per second screening, throughout.
- Ensure correct positioning of the subject throughout i.e. seat upright with a neutral head position.
- Ensure no use of swallowing strategies throughout e.g. head turns.

*Specified Contrast Media*

- The low osmolarity contrast media solution specified by Phagenesis will be used as the radiopaque contrast media during the STEPS VFS procedure.
- All volumes of contrast media will be measured accurately using a syringe.

*VFS Protocol (see also appendix M):*

- 6 trials of 5ml contrast media will be given to the subject from a small green Kapitex cup.
- Subjects will be asked to pour all of the contrast media into their mouth and then asked to swallow.
- 50ml contrast media will then be given in a ‘normal’ beaker and the subject asked to drink this sequentially.

*Criteria to Stop the STEPS VFS Protocol:*

Stop the VFS procedure immediately if any of the following occur:

Stage 1 (5ml trials)

- A PAS of 7 or 8 on 3 consecutive *bolus* trials.

Stage 2 (50ml trial)

- PAS score of 7 or 8 on 3 consecutive *swallows*.
- Initial occurrence of aspiration of more than 50% of the total bolus.

A ‘Stop’ may also be applied if 3 bolus trials have been complete and in the opinion of the staff conducting the VFS, the subject is unusually or unreasonably distressed, or if the subject becomes too unwell to continue.

A ‘Stop’ will NOT exclude the subject from the study.

VFS staff are asked to carry out the following actions in the event of the use of the stop criteria and/or significant aspiration.

- Prompt the subject to cough
- Prompt the subject to cough and swallow
- Refer the subject for chest physiotherapy as per local practice
- Arrange for the subject to be monitored over the next 24 hours as per local practice

The VFS images will be analysed off line by an independent panel of specialist SALTs who will respond with confirmation of the need for randomisation within 24 hours of screening VFS submission.

**Outcome scales**

***Penetration aspiration scale*** [***^3^***](#_ENREF_3)

| **Score** | **Description on videofluoroscopy** |
| --- | --- |
| 1 | Material does not enter airway |
| 2 | Material enters airway.  Remains above vocal cords & is ejected from airway |
| 3 | Material is above vocal cords & is not ejected from airway |
| 4 | Material enters airway, contacts vocal cords & ejected from airway |
| 5 | Material contacts the vocal cords & is not ejected from airway |
| 6 | Material passes below the vocal cords & is ejected into larynx or out of airway |
| 7 | Material passes below the vocal cords & is not ejected from the trachea despite effort |
| 8 | Material enters airway, passes below the vocal cords & no effort is made to eject the material |

***Dysphagia severity rating scale (DSRS)*** [^2^](#_ENREF_2)

DSRS is a derivative of the dysphagia outcome and severity scale.[^4^](#_ENREF_4)

| **Score** | **Fluids** | **Score** | **Diet** | **Score** | **Supervision** |
| --- | --- | --- | --- | --- | --- |
| 4 | No oral fluids | 4 | Non oral feeding | 4 | No oral feeding |
| 3 | Pudding consistency | 3 | Puree | 3 | Therapeutic feeding  (SALT/trained staff) |
| 2 | Custard consistency | 2 | Soft, moist diet | 2 | Feeding by third party  (untrained) |
| 1 | Syrup consistency | 1 | Selected textures | 1 | Eating with supervision |
| 0 | Normal fluids | 0 | Normal | 0 | Eating independently |

**Statistics**

Since a treatment could, in principle, be associated both with improved outcome and death, a sensitivity analysis was performed with death assigned a score one worse than the worst possible PAS (death = 9) and DSRS (13) scores; this is analogous to the mRS and EQ-5D-HUS which both include death in their scores (6 and 0 respectively).

**Role of the funding source**

The trial was overseen by the Chief Investigator (PB), PES Inventor (SH), and Trial Manager (JL), and run by the Trial Management Committee (PMB, SH, CM, JL), this including senior representatives of the funding and sponsoring company, Phagenesis Ltd (CM, JL). Sites received regular monitoring with 100% data verification. Data were collected using a commercial database (Rave, Medidata Solutions Inc.). Analyses were performed by PS at the University of Nottingham (PS). Interpretation and report writing were performed by the Trial Management Committee and National Coordinating Investigators. The corresponding author and another author (statistician PS) had full access to all the data in the study; additionally, the corresponding author had final responsibility for the decision to submit for publication, and is the guarantor for the study.

**Amendments to clinical investigation**

| Version: date | Reason for Amendment | Countries affected |
| --- | --- | --- |
| Final: 15/11/2011 | Not actively used by sites – see amendment 1 | France  UK |
| 1: 30/1/2012 | - At request of UK REC - to include statement that clinical decision would take precedent - Clarify what will happen to data after 15 years | France  UK |
| 2: 19/9/2012 | Non-substantial amendment:   - Amendment to statement of conformity as now CE marked device. - Addition to the secondary objectives - Increased length of investigation to 18 months - Removed minimum and maximum site recruitment numbers - Increase in the number of sites - Clarification on treatment of patients according to local best clinical practice - Clarification of wording for VFS procedure   Substantial amendment:   - Addition to the secondary endpoints - Amendment to consent process at investigational sites in Germany - Addition of code-break procedure - Addition of safety reporting responsibilities | Denmark  France  Germany  Spain  UK |
| 3: 6/6/2013 | Non-substantial amendment:   - Increased length of investigation - Increased number if investigation sites - Informed consent in non-UK sites to follow local practice and in line with country approvals. | Denmark  France  Germany  Spain  UK |
| 4: 21/1/2014 | Non-substantial amendment:   - Increased length of investigation - Increased number of subjects from 140 to 160 - Clarify target population - Clarify speech & language therapist plan | Denmark  France  Germany  Spain  UK |

VFS: videofluoroscopy

**SUPPLEMENTAL TABLES**

**Supplementary Table I**. Treatment dose and tolerance in 141 participants who received at least one treatment session. Treatment level is that actually received by patients randomised to pharyngeal electrical stimulation, or what patients randomised to sham would have received if actively treated.

|  | All | PES | Sham |
| --- | --- | --- | --- |
| Patients | 141 | 78 | 63 |
| Number of treatments | 414 | 233 | 181 |
| No. of treatments per patient | 2.9 | 3.0 | 2.9 |
| Threshold level (mA) | 9.5 (6.2) | 8.9 (5.1) | 10.3 (7.4) |
| Range | 1, 47 | 1, 30 | 1, 47 |
| Tolerance level (mA) | 16.9 (9.2) | 16.7 (8.9) | 17.3 (9.7) |
| Range | 3, 50 | 4, 50 | 3, 50 |
| Treatment level (mA) | 14.8 (7.9) | 14.5 (7.5) | 15.1 (8.3) |
| Range | 2, 50 | 2, 45 | 2, 50 |
| Treatment – threshold (mA) | 5.3 (5.3) | 5.6 (5.6) | 4.9 (5.0) |
| Range | -17, 27 | -2, 27 | -17, 26 |
| Duration (minutes) | 9.9 (1.2) | 9.8 (1.4) | 10.0 (0.7) |
| Range | 0, 10 | 0, 10 | 0.2, 10 |
| Undertreated (%) † | 85 (60.7) | 45 (58.4) | 40 (63.5) |

† Under-treatment is defined as patients with treatment <7.3 mA (mean - 1 standard deviation [^5^](#_ENREF_5)) or treatment-threshold level <=0 mA.

**Supplementary Table II.** Penetration aspiration score (PAS), clinical and safety outcomes by treatment assignment (intention-to-treat) in patients who received at least one active or sham treatment and who had outcome measured. Data are number (%), median [interquartile range], or mean (standard deviation). Comparisons using multiple linear regression, ordinal logistic regression, Cox regression or binary logistic regression. Analyses were adjusted for site, age, NIHSS, feeding status, baseline PAS and baseline value; or unadjusted.

|  | N | All | PES | Sham | OR/HR/MD | 2p adjusted | OR/HR/MD | 2p unadjusted |
| --- | --- | --- | --- | --- | --- | --- | --- | --- |
| *PAS sensitivity analyses* |  |  |  |  |  |  |  |  |
| Mean of all boli (/9) † | 126 | 3.7 (2.1) | 3.7 (2.1) | 3.7 (2.0) | 0.24 (-0.32, 0.79) | 0.40 | 0.04 (-0.67, 0.75) | 0.91 |
| Change ‡ | 123 | -1.1 (1.8) | -1.2 (1.8) | -1.1 (1.8) | 0.05 (-0.45, 0.54) | 0.86 | -0.05 (-0.67, 0.58) | 0.89 |
| Mean of all swallows (/8) | 126 | 3.2 (1.8) | 3.1 (1.8) | 3.2 (1.8) | 0.08 (-0.35, 0.50) | 0.73 | -0.07 (-0.70, 0.55) | 0.82 |
| Mean number of swallows | 126 | 16.2 (8.7) | 16.4 (9.9) | 16.0 (7.0) | -0.23 (-2.66, 2.21) | 0.86 | 0.43 (-2.62, 3.47) | 0.78 |
| Mean of first 3 boli (/8) | 126 | 3.1 (2.2) | 3.1 (2.2) | 3.1 (2.2) | 0.32 (-0.29, 0.92) | 0.31 | 0.03 (-0.74, 0.79) | 0.95 |
| % of boli >3 | 126 | 40.0 (31.7) | 39.6 (31.8) | 40.4 (31.7) | 3.47 (-8.14, 15.07) | 0.56 | 2.06 (-11.66, 15.78) | 0.77 |
| Worst score (/8) | 126 | 8 [5, 8] | 8 [6, 8] | 8 [4.5, 8] | 1.99 (0.83, 4.78) | 0.12 | 1.68 (0.84, 3.35) | 0.14 |
| *2 weeks* |  |  |  |  |  |  |  |  |
| TOR-BSST, failed (%) | 127 | 113 (89.0) | 62 (88.6) | 51 (89.5) | 0.67 (0.08, 5.39) | 0.71 | 0.91 (0.30, 2.80) | 0.87 |
| Feeding (%) | 132 |  |  |  | 2.07 (0.97, 4.39) | 0.059 | 1.42 (0.77, 2.63) | 0.27 |
| Oral, normal food |  | 27 (20.5) | 17 (23.3) | 10 (16.9) |  |  |  |  |
| Oral, soft diet |  | 44 (33.3) | 16 (21.9) | 28 (47.5) |  |  |  |  |
| Nasogastric tube |  | 31 (23.5) | 21 (28.8) | 10 (16.9) |  |  |  |  |
| PEG |  | 11 (8.3) | 7 (9.6) | 4 (6.8) |  |  |  |  |
| Other |  | 19 (14.4) | 12 (16.4) | 7 (11.9) |  |  |  |  |
| Weight (kg) | 129 | 71.2 (15.3) | 71.0 (14.7) | 71.4 (16.1) | 0.49 (-0.72, 1.69) | 0.43 | -0.34 (-5.63, 4.94) | 0.90 |
| BMI (kg/m^2^) | 127 | 25.0 (4.8) | 25.4 (4.4) | 24.4 (5.1) | 0.23 (-0.20, 0.66) | 0.29 | 1.00 (-0.64, 2.65) | 0.23 |
| MAC (m) | 129 | 28.4 (4.2) | 28.3 (3.7) | 28.5 (4.8) | 0.20 (-0.72, 1.11) | 0.68 | -0.22 (-1.66, 1.22) | 0.77 |
| Albumin (g/l) | 105 | 36.8 (5.3) | 37.0 (5.7) | 36.6 (4.8) | -0.16 (-1.39, 1.08) | 0.80 | 0.37 (-1.70, 2.44) | 0.72 |
| *12 weeks* |  |  |  |  |  |  |  |  |
| TOR-BSST, failed (%) | 103 | 75 (72.8) | 42 (72.4) | 33 (73.3) | 0.88 (0.17, 4.41) | 0.87 | 0.95 (0.40, 2.29) | 0.92 |
| HADS (/42) | 92 | 11.5 (7.4) | 11.0 (6.7) | 12.1 (8.2) | 1.80 (-0.97, 4.58) | 0.20 | -1.07 (-4.07, 1.93) | 0.49 |
| Weight (kg) | 101 | 73 (14.9) | 72.1 (14.3) | 74.1 (15.8) | 0.56 (-1.02, 2.14) | 0.49 | -1.97 (-7.80, 3.85) | 0.51 |
| BMI (kg/m^2^) | 98 | 25.5 (4.6) | 25.6 (4.2) | 25.4 (5.0) | 0.37 (-0.19, 0.94) | 0.20 | 0.25 (-1.56, 2.06) | 0.79 |
| MAC (m) | 104 | 28.3 (3.6) | 27.9 (3.5) | 28.7 (3.7) | -0.05 (-0.83, 0.72) | 0.89 | -0.79 (-2.18, 0.60) | 0.27 |
| Albumin (g/l) | 62 | 41.4 (5.2) | 41.7 (4.6) | 41.1 (6.0) | -0.28 (-1.83, 1.27) | 0.73 | 0.63 (-1.96, 3.23) | 0.63 |
| Disposition (%) | 141 |  |  |  | 0.66 (0.30, 1.49) | 0.32 | 0.63 (0.31, 1.26) | 0.19 |
| Home |  | 30 (21.3) | 20 (25.6) | 10 (15.9) |  |  |  |  |
| Institution |  | 93 (66.0) | 49 (62.8) | 44 (69.8) |  |  |  |  |
| Died |  | 18 (12.8) | 9 (11.5) | 9 (14.3) |  |  |  |  |

† Includes death: PAS=9; ‡ All treatments received (N=123)

BMI: body mass index; MAC: mid arm circumference; TOR-BSST: Toronto Bedside Swallowing Screening Test

**Supplementary Table III.** Participants in the safety population (N=152) with one or more serious adverse events (SAE) up to 12 weeks; no serious adverse device effects (SADE) occurred. Data are number (%) for total and fatal events. Comparison by unadjusted binary logistic regression.

|  |  | Any |  |  |  | Fatal |  |  |
| --- | --- | --- | --- | --- | --- | --- | --- | --- |
|  | All | PES | Sham | P | All | PES | Sham | p |
| Patients | 152 | 85 | 67 |  | 152 | 85 | 67 |  |
| Cardiac | 9 (5.9) | 6 (7.1) | 3 (4.5) | 0.73 | 4 (2.6) | 2 (2.4) | 2 (3.0) | 1.00 |
| Gastrointestinal | 2 (1.3) | 2 (2.4) | 0 (0) | 0.50 | 0 (0) | 0 (0) | 0 (0) | - |
| General | 3 (2.0) | 0 (0) | 3 (4.5) | 0.083 | 3 (2.0) | 0 (0) | 3 (4.5) | 0.083 |
| Hepatobiliary | 1 (0.7) | 1 (1.2) | 0 (0) | 1.00 | 0 (0) | 0 (0) | 0 (0) | - |
| Infections | 11 (7.2) | 6 (7.1) | 5 (7.5) | 1.00 | 4 (2.6) | 2 (2.4) | 2 (3.0) | 1.00 |
| Investigations | 1 (0.7) | 1 (1.2) | 0 (0) | 1.00 | 0 (0) | 0 (0) | 0 (0) | - |
| Neoplasms | 1 (0.7) | 1 (1.2) | 0 (0) | 1.00 | 1 (0.7) | 1 (1.2) | 0 (0) | 1.00 |
| Nervous system | 8 (5.3) | 4 (4.7) | 4 (6.0) | 0.73 | 4 (2.6) | 3 (3.5) | 1 (1.5) | 0.63 |
| Renal/urinary | 2 (1.3) | 1 (1.2) | 1 (1.5) | 1.00 | 0 (0) | 0 (0) | 0 (0) | - |
| Respiratory | 8 (5.3) | 5 (5.9) | 3 (4.5) | 1.00 | 2 (1.3) | 1 (1.2) | 1 (1.5) | 1.00 |
| Surgical/medical | 2 (1.3) | 2 (2.4) | 0 (0) | 0.50 | 0 (0) | 0 (0) | 0 (0) | - |
| Total SAEs | 40 (26.3) | 22 (25.9) | 18 (26.9) | 1.00 | 18 (11.8) | 9 (10.6) | 9 (13.4) | 0.62 |
| Total SADEs | 0 (0) | 0 (0) | 0 (0) |  | 0 (0) | 0 (0) | 0 (0) |  |

By 2 weeks, SAE rates were: Total 13 (9.2%), PES 7 (9.0%), Sham 6 (9.5%) (2p=0.91)

No SAEs were recorded as probable or possibly related to treatment

No Serious Adverse Device Effects (SADE) were recorded

**Supplementary Table IV**. Treatment operator assessment of ease of use of device in 162 randomised patients across both treatment groups. Data are numbers of patients (%).

|  | Difficult |
| --- | --- |
| Provision of treatment | 3 (2.2) |
| Meet infection control guidelines | 0 (0) |
| Placement of catheter | 10 (7.5) |
| Passing catheter (as NGT) | 44 (33.1) |
| Secure catheter | 19 (14.3) |
| Training to deliver treatment | 3 (2.7) |

**Supplementary Table V**. Penetration aspiration score (PAS) and dysphagia severity rating scale (DSRS) before and after treatment in the active and sham groups in four trials of pharyngeal electrical stimulation. Patients with both baseline and 2 week mean PAS are included. Data are unadjusted PAS and DSRS mean scores or differences.

| Group | Timing |  | PAS |  |  |  | DSRS |  |  |
| --- | --- | --- | --- | --- | --- | --- | --- | --- | --- |
|  |  | Trial 1 [^2^](#_ENREF_2) | Trial 2 [^2^](#_ENREF_2) | Trial 3 [^6^](#_ENREF_6) | STEPS | Trial 1 [^2^](#_ENREF_2) | Trial 2 [^2^](#_ENREF_2) | Trial 3 [^6^](#_ENREF_6) | STEPS |
| Active | Before | 4.8 | 4.6 | 4.5 | 4.8 | ND | 6.4 | 7.8 | 7.7 |
|  | After | 3.7 | 3.2 | 2.6 | 3.6 | ND | 2.5 | 4.4 | 5.0 |
|  | ∆ | -1.0 | -1.4 | -1.9 | -1.1 | ND | -3.9 | -3.4 | -2.7 |
| Sham | Before | 4.3 | 3.9 | 3.9 | 4.7 | ND | 5.6 | 6.8 | 6.8 |
|  | After | 4.8 | 3.8 | 4.3 | 3.6 | ND | 4.8 | 5.0 | 4.8 |
|  | ∆ | +0.5 | -0.1 | +0.4 | -1.1 | ND | -0.8 | -1.8 | -2 |
| Active-sham | ∆ | -1.5 | -1.3 | -2.3 | 0 | ND | -3.0 | -1.5 | -0.6 |
|  | p | 0.017 | 0.061 | 0.22 | 1.00 | ND | 0.11 | 0.11 | 0.25 |

ND: Not done

**SUPPLEMENTAL FIGURES**

**Supplementary Figure I.** Effect of treatment on dysphagia severity rating scale in 131 patients at 2 weeks in pre-specified subgroups determined at baseline. Analysed with multiple linear regression adjusted for baseline PAS, stratification variables (site, feeding status) and prognostic baseline variables (age, baseline PAS, NIHSS).


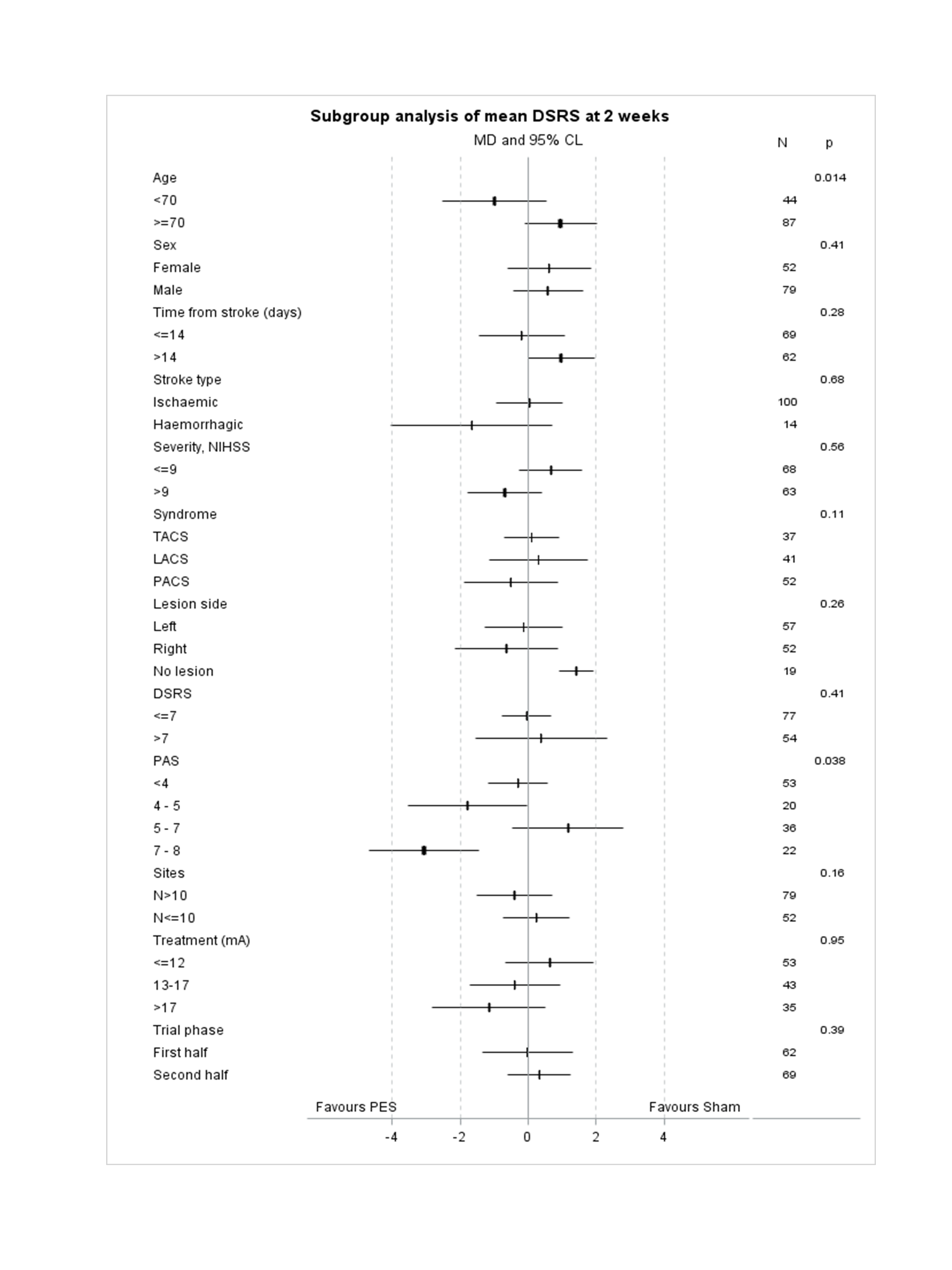


The black squares represent point estimates for the mean difference, and the horizontal lines represent 95% confidence intervals. The rectangle incorporates the point estimate and the 95% confidence intervals of the overall effects within categories. P values are for the interaction between subgroup and allocated treatment.

**Supplementary Figure II**. Survival of patients randomised to pharyngeal electrical stimulation (PES) versus sham. Adjusted hazard ratio 1.11 (0.34,3.59), p=0.86 (n=141).


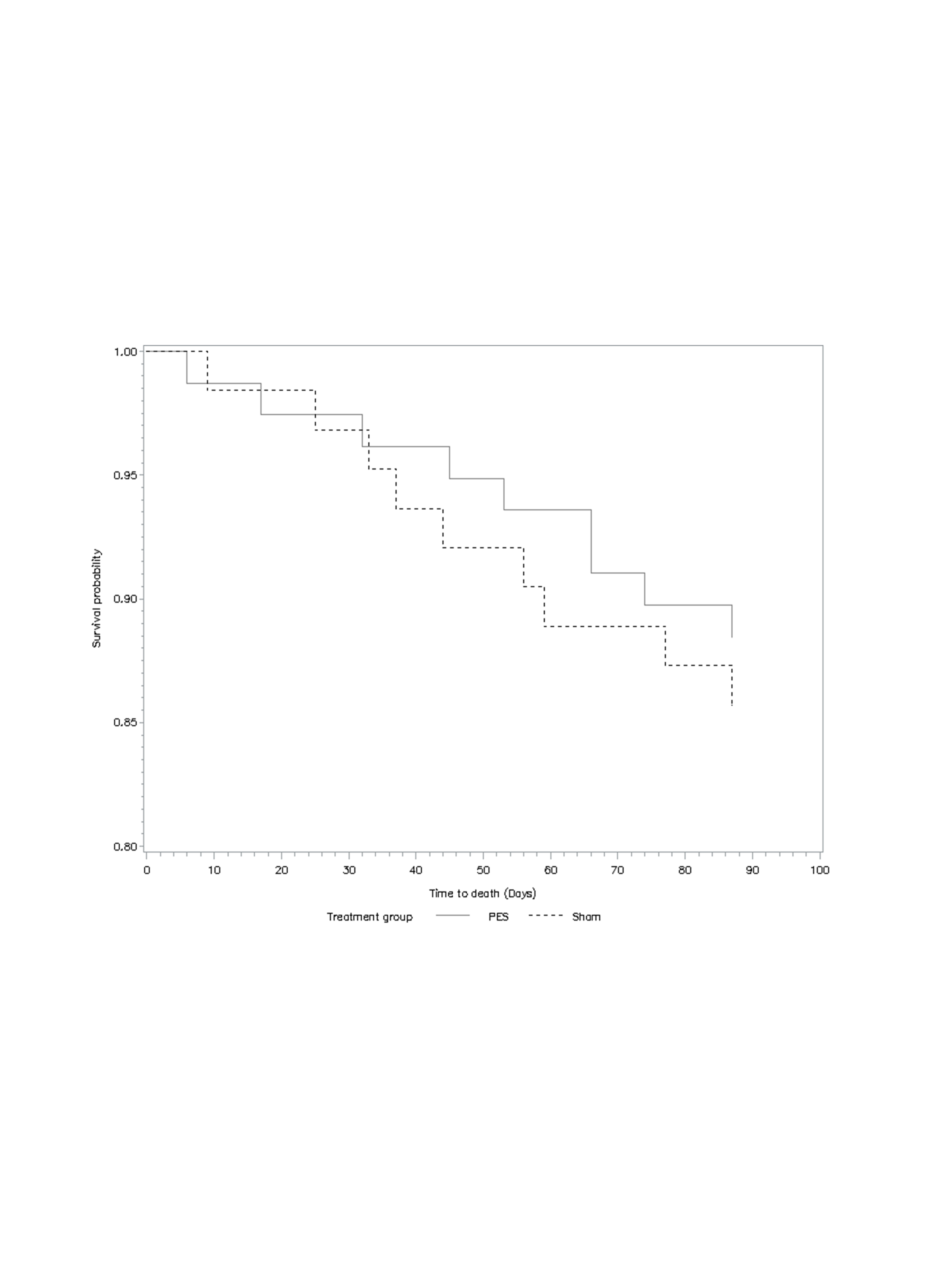


**Supplementary Figure III**. Meta-analysis of the effect of pharyngeal electrical stimulation (PES) on radiological aspiration at two weeks, as assessed using the penetration aspiration score (PAS). Three previous trials and the present trial are included. Data are mean change in PAS, with 95% confidence intervals, using a random effects model.


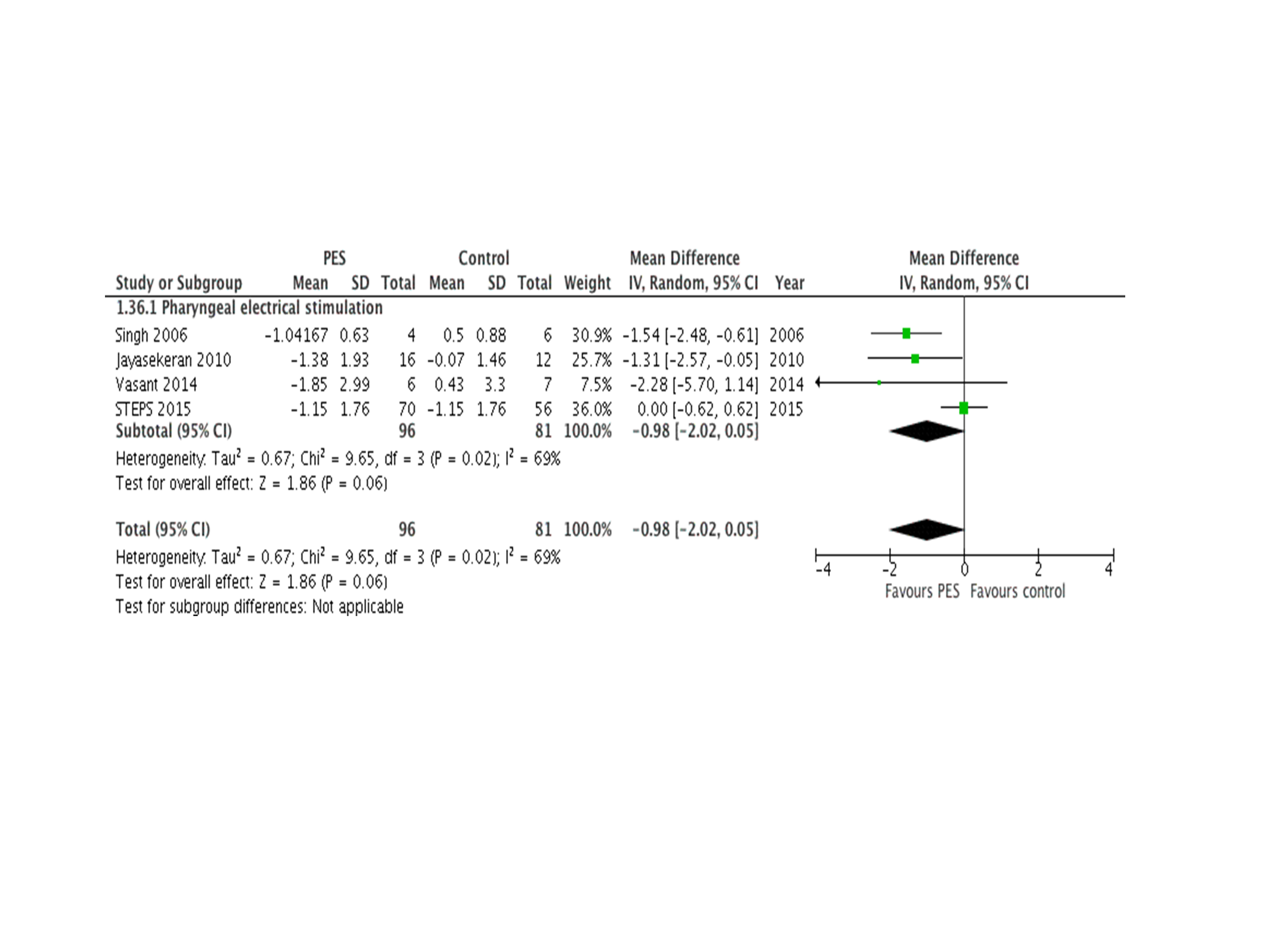


**Supplementary Figure IV**. Meta-analysis of the effect of pharyngeal electrical stimulation (PES) on clinical dysphagia at two weeks, as assessed using the dysphagia severity rating scale (DSRS). Two previous trials and the present trial are included. Data are mean change in DSRS from baseline, with 95% confidence intervals, using a random effects model.


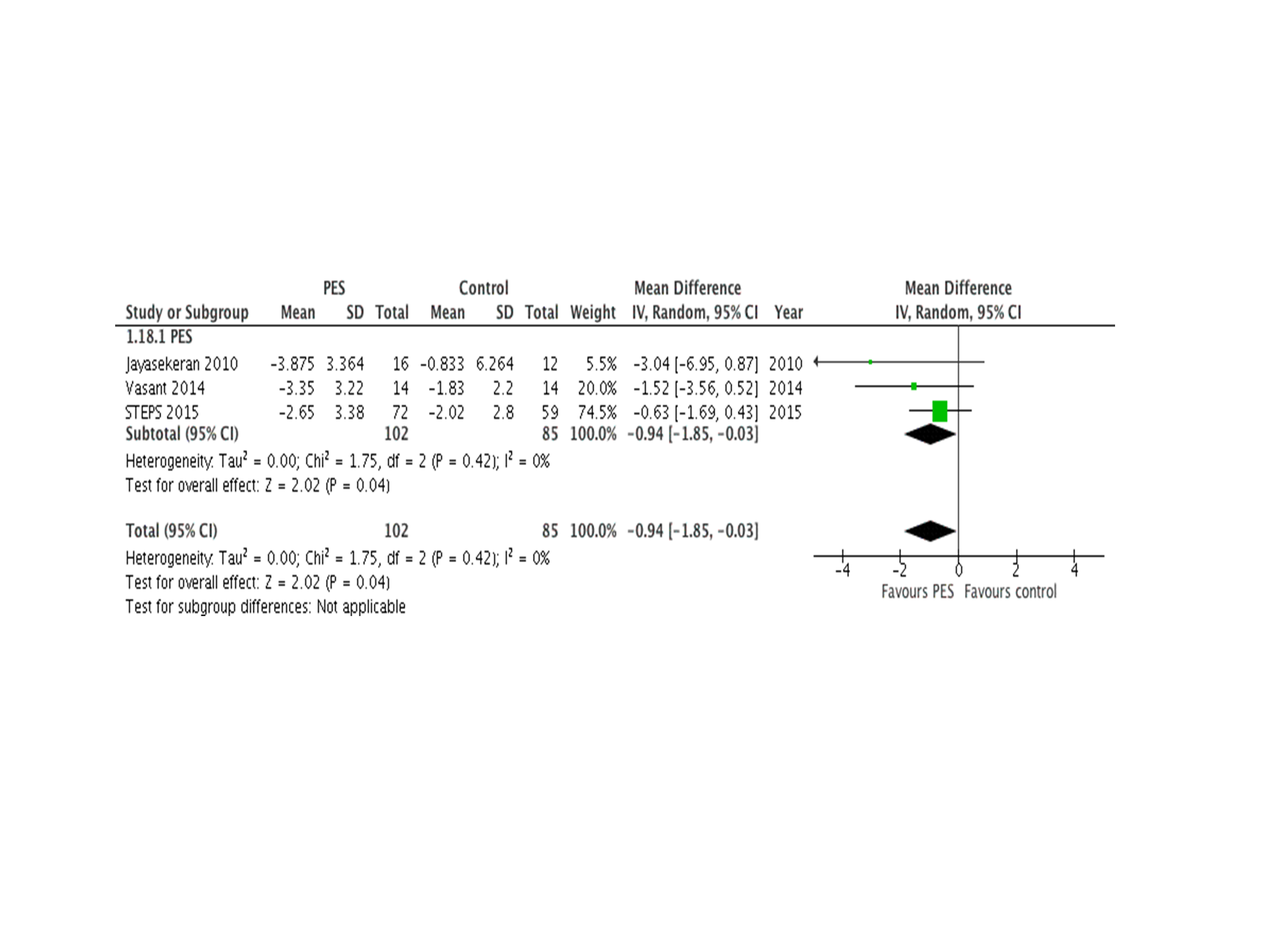


**REFERENCES**

1. Buyse M, George SL, Evans S, Geller NL, Ranstam J, Scherrer B, et al. The role of biostatistics in the prevention, detection and treatment of fraud in clinical trials. *Stat Med*. 1999;18:3435-3451

2. Jayasekeran V, Singh S, Tyrrell P, Michou E, Jefferson S, Mistry S, et al. Adjunctive functional pharyngeal electrical stimulation reverses swallowing disability after brain lesions. *Gastroenterology*. 2010;138:1737-1746

3. Rosenbek J, Robbins J, Roecker E, Coyle J, Wood J. A penetration-aspiration scale. *Dysphagia*. 1996;11:93-98

4. O'Neil K, Purdy M, Falk J, Gallo L. The dysphagia outcome and severity scale. *Dysphagia*. 1999;14:139-145

5. Bath P, Scutt P, Hamdy S. Electrical pharyngeal stimulation for post stroke dysphagia: A meta-analysis of individual patient data from randomized controlled trials. *International Journal of Stroke*. 2014;9:103

6. Vasant D, Michou E, Tyrrell P, Jayasekeran V, Mistry S, O'Leary N, et al. Oc-063 pharyngeal electrical stimulation (pes) in dysphagia post-acute stroke: A double blind, randomised trial. *Gut*. 2014;63
